# Effect of Cu Content on Corrosion Resistance of 3.5%Ni Weathering Steel in Marine Atmosphere of South China Sea

**DOI:** 10.3390/ma18153496

**Published:** 2025-07-25

**Authors:** Yuanzheng Li, Ziyu Guo, Tianle Fu, Sha Sha, Bing Wang, Xiaoping Chen, Shujun Jia, Qingyou Liu

**Affiliations:** 1 Engineering Steel Research Institute, Central Iron and Steel Research Institute Company Limited, Beijing 100081, China; yuan20084@163.com (Y.L.); 18332260911@163.com (Z.G.); 18574437760@163.com (T.F.); 15161021197@163.com (S.S.); xiaoping118@sina.com (X.C.); jiajsj504@126.com (S.J.); liuqingyou@nercast.com (Q.L.); 2 CNPC Baoji Petroleum Pipe Industry Company Limited, Baoji 721002, China; 3 College of New Energy and Materials, China University of Petroleum, Beijing 102249, China; 4 Suzhou Jiangnan Aerospace Mechanical & Electrical Industry Company, Suzhou 215000, China

**Keywords:** harsh oceanic atmosphere environment, dry–wet cycle accelerated corrosion test, 3.5%Ni weathering steel, Cu content

## Abstract

The influence of the copper (Cu) content on the corrosion resistance of 3.5%Ni low-carbon weathering steel was investigated using periodic dry–wet cycle accelerated corrosion tests. The mechanical properties of the steels were assessed via tensile and low-temperature impact tests, while corrosion resistance was evaluated based on weight loss measurements. Surface oxide layers were characterized using three-dimensional laser confocal microscopy, X-ray diffraction (XRD), X-ray photoelectron spectroscopy (XPS), and electrochemical methods. Electron probe microanalysis (EPMA) was employed to examine the cross-sectional morphology of the oxide layer after 72 h of accelerated corrosion tests. The results indicate that the solution state of Cu increased the strength of 3.5%Ni steels but significantly damaged the low-temperature toughness. As the Cu content increased from 0.75% to 1.25%, the corrosion rate decreased from 4.65 to 3.74 g/m^2^ h. However, when there was a further increase in the Cu content to 2.15%, there was little decrease in the corrosion rate. With the increase in the Cu content from 0.75% to 2.15%, the surface roughness of 3.5%Ni weathering steel after corrosion decreased from 5.543 to 5.019 μm, and the corrosion behavior was more uniform. Additionally, the α/γ protective factor of the oxide layer of the surface layer increased from 2.58 to 2.84 with an increase in the Cu content from 0.75% to 1.25%, resulting in the oxide layer of the surface layer being more protective. For 1.25%Cu steel, the corrosion current density of rusted samples is lower (ranging from 1.2609 × 10^−4^ A/cm^2^ to 3.7376 × 10^−4^ A/cm^2^), and the corrosion potential is higher (ranging from −0.85544 V to −0.40243 V). Therefore, the rusted samples are more corrosion resistant. The Cu in the oxide layer of the surface layer forms CuO and CuFeO_2_, which are helpful for increasing corrosion resistance, which inhibits the penetration of Cl^−^.

## 1. Introduction

Marine engineering equipment plays a vital role in deep-sea resource exploitation, offshore energy utilization, and national defense [1,2,3,4,5,6]. However, the unique marine atmospheric environment of the South China Sea—characterized by high salinity, humidity, temperature, and intense ultraviolet radiation—severely challenges structural material corrosion resistance [7,8]. Conventional corrosion-resistant steels often degrade during long-term service, compromising structural integrity and posing safety risks. Consequently, developing novel steels tailored to the South China Sea’s extreme conditions has become critical for marine engineering materials [9,10].

Traditional corrosion-resistant steels (e.g., carbon steels or low-alloy steels) typically form loose, porous oxide layers in marine environments, which inadequately block corrosive agent penetration [11]. While alloying elements like Cr and Mo enhance corrosion resistance, their high cost and adverse effects on workability limit their practical application [12,13]. Moreover, the complex South China Sea climate accelerates localized corrosion (e.g., pitting and crevice corrosion), significantly reducing equipment service life. There is consequently an urgent need to develop cost-effective, high-performance steels through composition optimization and microstructure control.

Recent studies highlight the role of Ni and Cu elements in weathering steels [14,15]. Ni stabilizes the α-FeOOH phase in the oxide layer of the surface layer, improving its compactness, while Cu facilitates the formation of a protective oxide layer at the surface layer to suppress corrosion propagation [16,17]. Ni can also improve the hot shortness phenomenon of Cu-bearing steel during hot rolling so that Cu would be completely dissolved in the hot-rolled austenite [18]. The corrosion inhibition mechanism of Cu depends on its chemical occurrence state (e.g., solid solution or precipitates): solid solution Cu enhances oxide layer conductivity, whereas nano-sized Cu-rich precipitates refine rust particles [19,20]. The solid solubility of Cu in steel is higher than that of other microalloying elements (such as Nb, V, Ti, etc.), thus facilitating the regulation of the occurrence of Cu in low-alloy high-strength steel [4,5,6,21]. The effective temperature range for the precipitation of Cu in steel is 400–600 °C [22,23]. The structure evolution of Cu-rich phase precipitation follows the following sequence: B2→BCC→9R→3R→FCC [24,25]. With the continuation of this evolution sequence, the misfit degree between the precipitation structure and the matrix gradually increases, resulting in a potential difference between the structure and the matrix, and then forming a corrosion couple, increasing the corrosion rate [20].

Therefore, in the two states where Cu elements are present, it is currently generally believed that the solid solution of Cu elements is beneficial to the improvement in corrosion resistance. The adjustment of Cu elements in steel is usually accompanied by changes in the microstructure. Zhang et al. regulated marine atmospheric corrosion-resistant steel containing 1.2%Cu by using water cooling and air cooling processes and systematically studied the influence of Cu on the microstructure and corrosion resistance [19]. The semi-coherent interface between ε-Cu precipitation and the matrix increased corrosion sensitivity and reduced the protective property of the oxide layer, while the solid solution Cu increased the corrosion potential and the content of CuFeO_2_ in the oxide layer of the surface layer. Therefore, protection was enhanced. Although present works focus on the effect of the occurrence of Cu on corrosion resistance performance, it is still necessary to quantify the influence relationship of the Cu content on corrosion resistance. Moreover, traditional studies focus on the effect of Cu precipitation rather than the complete solid solution on corrosion resistance. The synergistic effects of Cu in a solid solution state and the optimal Ni/Cu content under the highly aggressive South China Sea environment require systematic investigation.

The long-term corrosion resistance of weathering steels relies on the stability and self-repairing capacity of the oxide layer of the surface layer. In marine atmospheres, Cl^−^ infiltration disrupts the rust structure, but Ni and Cu promote the formation of a dense inner oxide layer at the surface layer (e.g., Fe_3_O_4_/α-FeOOH), inhibiting Cl^−^ diffusion [26,27,28]. Additionally, Cu segregation may generate Cu-O compounds, further passivating the corrosion reaction [19]. Thus, elucidating the relationship between the Ni/Cu content, rust composition, and microstructure is essential for designing advanced corrosion-resistant steels.

This study investigates the influence of the Ni/Cu contents and their chemical states on the formation of an oxide layer at the surface layer and corrosion behavior in the South China Sea marine environment. By combining microstructural characterization (SEM and XPS) with electrochemical tests, we reveal the corrosion inhibition mechanisms of key alloying elements and establish a composition–rust performance model. The findings will provide theoretical and technical guidance for material selection and the development of marine engineering steels.

## 2. Materials and Methods

### 2.1. Sample Preparation

The detected chemical compositions of three weathering steels are listed in Table 1. The main difference is seen in the content of Cu. The Cu content was increased to enhance the corrosion resistance performance. The rolling process was conducted as follows: Cast slabs (60 mm in thickness) were homogenized at 1200 °C for 1 h. The maximum rolling thickness of the experimental rolling mill was no more than 65 mm, so the thickness of 60 mm was selected for cast slabs. The homogenization process of 1200 °C for 1 h was used to weaken the segregation. Hot rolling was initiated at 1150 °C, and the final finishing pass was performed at 800 °C. After six rolling passes, steel plates with 7 mm thickness were obtained. A similar hot rolling process with a rolling compression ratio of ~88.3% was employed for the three tested steels to break coarse primary dendrites, make the steel denser, and refine the prior austenite grain size. In order to improve the comprehensive mechanical properties of Cu-bearing steel, a certain proportion of non-recrystallization rolling was employed. The temperature for effective Cu precipitation ranges from 400 to 600 °C. To achieve a bainitic microstructure and suppress Cu precipitation to give full play to corrosion resistance performance [29], the following controlled cooling strategy was employed: water quenching at 350 °C, followed by an isothermal holding process at 350 °C for 1 h in a furnace to simulate coiling, with subsequent furnace cooling to room temperature. At 350 °C isothermal holding, the bainite transformation of the three steels was induced, and the Cu was dissolved in the matrix.

### 2.2. Microstructural Analysis

Specimens from the three tested steels were sequentially ground with 150-, 320-, 600-, and 1000-grit sandpaper, followed by final polishing using diamond abrasive. Polished samples were etched ∼6 times with 10 vol% HNO_3_ + 90 vol% C_2_H_5_OH solution (∼0.5 s per etch), ultrasonically cleaned in ethanol, and dried under compressed air. Microstructural characterization was subsequently performed using optical microscopy (OM) (Olympus Corporation, Tokyo, Japan) and FEI Quanta 650 field emission scanning electron microscopy (SEM), (FEI Company, Hillsboro, OR, USA).

### 2.3. Mechanical Property Tests

Round-bar tensile specimens (Φ3 mm) and V-notched Charpy impact specimens (5 mm × 10 mm × 55 mm) were prepared. Three parallel specimens were tested for final impact value. Impact tests followed GB/T 229 [30] at both room temperature (25 °C) and −20 °C. Tensile tests were conducted according to National Standard GB/T 228.1 [31] using a GNT100 electronic tensile testing machine (Gangyan NCS Testing Technology Co., Ltd., Beijing, China).

### 2.4. Periodic Immersion Wet–Dry Cyclic Corrosion Tests

The influence of Cu contents on corrosion resistance was investigated using periodic immersion wet–dry cyclic corrosion tests, where the specimen dimensions were 60 mm × 40 mm × 4 mm. Before wet–dry cyclic corrosion tests, all samples were ultrasonically cleaned in ethanol, dried with cold air, and weighed, with five parallel specimens included in each test group. The test samples were placed in the periodic immersion dry–wet cycle accelerated corrosion test device to conduct simulated marine atmospheric corrosion tests. Key test parameters included a temperature range of 43–45 °C, relative humidity of 65–75% (RH), and a total cycle duration of 60 min with an immersion-to-drying ratio of 1:4. During the drying phase, the maximum surface temperature of the specimens reached 70 ± 10 °C. The immersion solution consisted of 2% NaCl, which was replenished daily with 500 mL of distilled water to ensure consistent electrolyte concentration. Following the accelerated corrosion tests, the specimens were treated with a pickling solution to remove corrosion products. The mixed pickling solution included 500 mL of 36.5% HCl, 500 mL of deionized water, 10 g of hexamethylenetetramine, and 3 g of benzotriazole. After pickling, the specimens were dried and reweighed. Surface roughness at the center of each specimen was estimated using laser confocal microscopy (Carl Zeiss, Oberkochen, Germany). The corrosion depth (*h*, mm) was calculated using the following cubic equation:

(1) $$W_{t}=\left[h^{3}-\left(a+b+c\right)\cdot h^{2}+\left(ab+bc+ac\right)\cdot h\right]\cdot \frac{W_{0}}{a\cdot b\cdot c}$$

where *W*_0_ represents the initial weight of the specimen (unit: g); *Wₜ* denotes the weight after pickling at time *t* (unit: g); *a*, *b*, and *c* correspond to the length, width, and height of the specimen (unit: mm), respectively; and *t* indicates the total exposure time (unit: *h*). This methodology provided a quantitative assessment of corrosion behavior under simulated marine atmospheric conditions.

### 2.5. Electrochemical Evaluation

The electrochemical behavior was assessed using a Princeton 273A three-electrode workstation (Princeton Applied Research (PAR), Oak Ridge, TN, USA). The working electrode consisted of corroded electrochemical specimens after cyclic wet–dry accelerated corrosion tests, with a platinum counter electrode and saturated calomel electrode (SCE) as reference. Tests were performed in 2.0 wt% NaCl solution at 35 ± 0.5 °C, spanning from −0.5 V to +0.5 V versus open-circuit potential (OCP) at 1 mV·s^−1^.

### 2.6. X-Ray Diffraction (XRD) Analysis

Oxide layer structure of rust powders was identified using a Rigaku Smart Lab XRD (Rigaku Corporation, Akishima, Japan). During tests, its voltage and current were 40 kV and 30 mA, respectively. The scanning speed was 2°/min, and the step size was 0.02°. The phase measurement data were analyzed and processed by using the MDI Jade 6.5 software.

### 2.7. X-Ray Photoelectron Spectroscopy (XPS) Analysis

The elemental valence state in the oxide layer of the surface layer was analyzed using a Thermo Fisher Scientific (Thermo Fisher Scientific Inc, Waltham, MA, USA) K-Alpha XPS system with Al Kα radiation, whose hv is 1486.68 eV. The chamber vacuum was maintained at 5 × 10^−10^ Pa with a 15 kV excitation voltage and a 10 mA filament current. Spectra were acquired at 50 eV pass energy with a 0.05 eV step size, accumulating 5–10 scans. Charge correction was carried out by referencing the C1s peak at 284.8 eV, followed by data processing using Avantage software 6.9.0.

## 3. Results and Discussion

### 3.1. Initial Microstructure and Mechanical Properties

**Figure 1:**
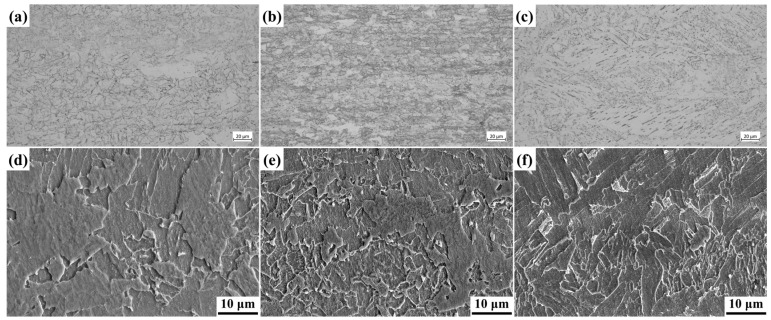
Microstructure characterizations: (**a**,**d**) 0.75Cu steel; (**b**,**e**) 1.25Cu steel; (**c**,**f**) 2.15Cu steel. (**a**–**c**) OM images. (**d**–**f**) SEM images.

Figure 1 presents the microstructure of Cu-bearing steels. The 0.75%Cu, 1.25%Cu, and 2.15%Cu steels exhibit bainitic matrix microstructures, with the 0.75%Cu variant containing a minor ferrite fraction. Microstructural refinement progresses with an increasing Cu content, attributable to copper’s austenite-stabilizing effect which enhances hardenability and lowers phase transformation temperatures [32]. Despite the low carbon content (∼0.02 wt%) in these 3.5%Ni weathering steels, the synergistic alloying effects of Ni-Cu-Mo ensure sufficient hardenability for bainite and martensite/austenite (M/A) constituent formation. Both the size and volume fraction of M/A constituents increase progressively with Cu addition, indicating decelerated phase transformation kinetics at higher copper concentrations.

The mechanical properties are listed in Table 2. For 0.75%Cu steel, the yield strength and tensile strength are approximately 560 and 680 MPa, respectively. There was little difference in impact energy at room temperature at 99 J and at −20 °C at 96 J. For 1.25%Cu steel, the yield strength rose to 651 MPa, and the tensile strength reached about 724 MPa, representing increases of 91 MPa and 44 MPa, respectively. The impact energies at room temperature and −20 °C were 104 J and 68 J, showing an increase of 5 J and a decrease of 28 J, respectively. The result implies that the ductile-to-brittle transition temperature (DBTT) of 0.75%Cu steel was lower than that of 1.25%Cu steel. For 2.15%Cu steel, the yield strength was 713 MPa, and the tensile strength was 822 MPa, representing increments of 62 MPa and 98 MPa, respectively. The impact energies at room temperature and −20 °C dropped to 25 J and 13 J, respectively. Compared to 0.75%Cu and 1.25%Cu steels, the DBTT of 2.15%Cu steel further increased, indicating that an increased Cu content deteriorates the toughness of HSLA steel.

The low Cu content, the fine size and low content of M/A island constituents, and a certain proportion of ferrite jointly achieve the high toughness of HSLA steel. Additionally, Cu is an element that is prone to segregation on grain boundary [33]. As is well known, Cu segregation can lead to a decline in impact energy. During controlling cooling, since the precipitation temperature range of Cu is entirely bypassed, all Cu in 3.5%Ni weathering steels remains in solid solution state. At the isothermal holding temperature of 350 °C, austenite transforms into bainite. At this temperature, Cu precipitation is difficult, but Cu segregation still occurs. The segregation of Cu significantly deteriorates low-temperature toughness.

### 3.2. Analysis of Corrosion Resistance Mechanisms

#### 3.2.1. Accelerated Corrosion Rate Analysis

**Figure 2:**
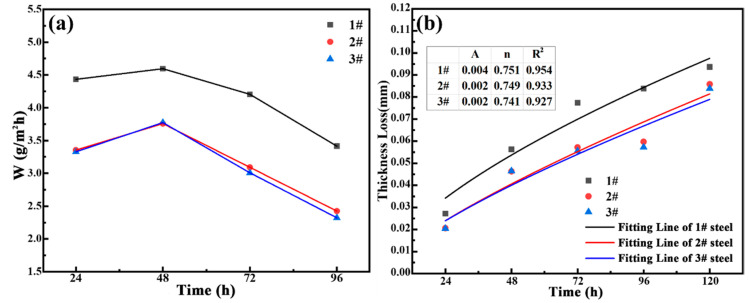
(**a**) Corrosion rate and (**b**) thickness loss of three 3.5%Ni weathering steels under different test cycles.

Figure 2a shows corrosion rate graphs of the test steel undergoing 24, 48, 72, and 96 h weekly immersion corrosion tests. The results show that the corrosion rates of all three test steels reach the maximum values of 4.65, 3.74, and 3.76 g/m^2^ h during 48 h of immersion corrosion for 0.75%Cu, 1.25%Cu, and 2.15%Cu steel. This is because in the early stage of corrosion, pitting corrosion is dominant, and a complete and dense rust layer has not been formed, providing relatively weak protection for the substrate. After the 48 h test period, the corrosion rate showed a downward trend. It was speculated that a protective layer capable of preventing corrosive substances from entering was produced, namely the formation of a rust layer. The corrosion rates of the three test steels were different. The corrosion rate of 0.75%Cu steel was higher than that of 1.25%Cu and 2.15%Cu steels, while the corrosion rates of the latter two basically coincided. By comparing the corrosion rates of 0.75%Cu steel and 1.25%Cu steel, it was confirmed that an increase in the copper content can enhance the corrosion resistance of 3.5%Ni weathering steel and lower the corrosion rate. By comparing the corrosion rates of 1.25%Cu and 2.15%Cu steels, it is indicated that further increasing the Cu content has little effect on corrosion resistance.

To evaluate the influence of Cu on the corrosion kinetics of 3.5%Ni weathering steel in a simulated tropical marine atmosphere, the thickness loss was fitted using the classical atmospheric corrosion function model [34]:

(2) $$C=A\cdot t^{n}$$

*C* represents the predicted value of corrosion weight loss; *t* represents the corrosion time, a; *A* and *n* are constants. *A* is generally the corrosion loss weight of the first a, which is related to the environment and material. The magnitude of n can reflect the protective effect of corrosion products on the material: when n > 1, the corrosion rate gradually increases. When n = 1, the corrosion rate remains unchanged. When n < 1, the corrosion rate gradually decreases. The fitted curves are shown in Figure 2b. Since all n values are below 1, three Cu-bearing steels have a low corrosion rate, potentially representing excellent corrosion resistance. The goodness of fit, R^2^, is high for all three curves of 3.5%Ni weathering steels, demonstrating high fitting accuracy. As the Cu content increases, both A and n decrease, further confirming that a higher Cu content enhances resistance to marine atmospheric corrosion of 3.5%Ni weathering steels. This result aligns well with subsequent electrochemical test results.

#### 3.2.2. Macroscopic Corrosion Morphology

**Figure 3:**
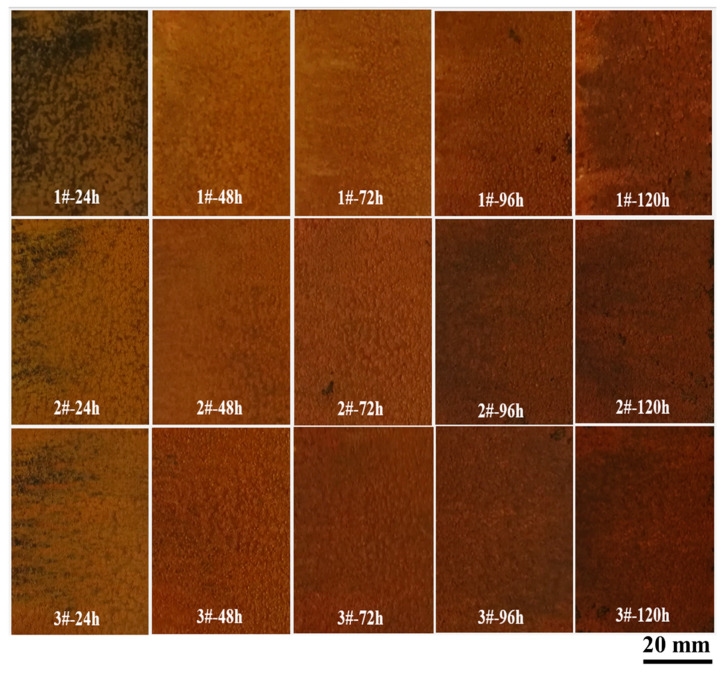
Corrosion morphology macroscopic images of three 3.5%Ni weathering steels under different test cycles (1#, 2#, and 3# are 0.75Cu, 1.25Cu, and 2.15Cu steels, respectively).

Figure 3 displays macroscopic morphology images of three Cu-bearing 3.5%Ni weathering steels after periodic immersion wet–dry cycle accelerated corrosion tests conducted over 24 h, 48 h, 72 h, 96 h, and 120 h periods. The results reveal uniformly distributed surface oxide layers exhibiting progressive darkening with exposure duration. At equivalent corrosion times, a higher Cu content correlates with increased reddish-brown coloration. Notably, during rust removal, steels with an elevated Cu content demonstrated greater oxide layer cohesion and markedly enhanced oxide–substrate adhesion, evidenced by increased resistance to mechanical removal.

#### 3.2.3. Surface Roughness (Sa) Analysis

**Figure 4:**
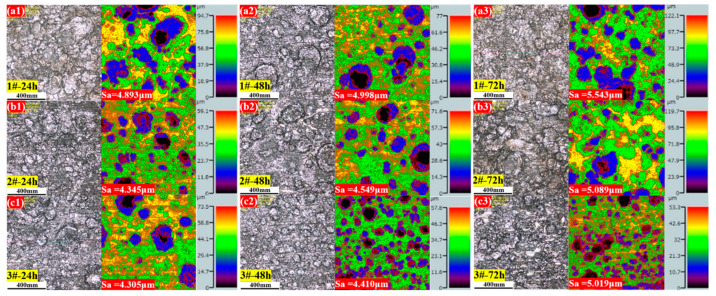
Macroscopic corrosion morphology and two-dimensional depth map of three Cu-bearing 3.5%Ni weathering steels subjected to wet–dry cycle accelerated corrosion tests: (**a1**–**a3**) 0.75Cu steel subjected to 24 h, 48 h, and 72 h tests, respectively; (**b1**–**b3**) 1.25Cu steel subjected to 24 h, 48 h, and 72 h tests, respectively; (**c1**–**c3**) 2.15Cu steel subjected to 24 h, 48 h, and 72 h tests, respectively.

Sa reflects the surface roughness of the substrate after corrosion. A higher Sa value on the corrosion surface indicates a more severe degree of corrosion behavior, whereas a lower Sa value corresponds to reduced corrosion damage in three Cu-bearing 3.5%Ni weathering steels. The same rule regarding Sa and corrosion-resisting properties has been confirmed in other studies [35,36]. A three-dimensional laser confocal microscope was employed to characterize the corrosion depth of three Cu-bearing 3.5%Ni weathering steels subjected to varying corrosion durations, as shown in Figure 4. As illustrated in Figure 4(a1), the left image is the macroscopic morphology after acid washing following corrosion, the middle image is the contour of the corrosion depth after acid washing, and the right image is the corrosion depth corresponding to the color of the contour lines.

Progressive corrosion testing revealed an increasing corrosion pit size/number density accompanied by decreasing Sa values. Simultaneously, higher Cu contents yielded progressively lower Sa values and greater pit density, indicating enhanced corrosion resistance in 3.5%Ni weathering steel through reduced localized attack severity. This improved corrosion uniformity promotes homogeneous surface oxide layer formation—a prerequisite for developing dense, protective rust structures.

#### 3.2.4. Element Distribution in Oxide Layer of Surface Layer

**Figure 5:**
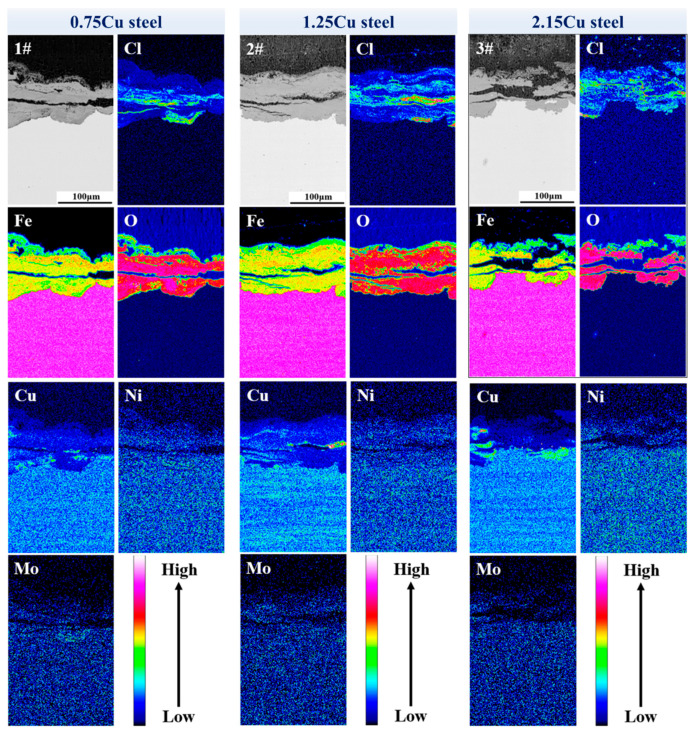
EPMA images showing element distributions of oxide layer of surface layer subjected to accelerated dry–wet cycle corrosion tests for 72 h.

An analysis of element distribution in the oxide layer of the surface layer is helpful for estimating the corrosion resistance mechanism. The distribution of Cl, Cu, Ni, Fe, O, and Mo elements in the cross-section of the oxide layer of the surface layer in three Cu-bearing 3.5%Ni weathering steels after 72 h of periodic immersion and wet–dry cycle accelerated corrosion tests is presented in Figure 5. Significant heterogeneity was observed in the distribution of Fe and O elements, particularly showing localized regions with markedly higher or lower Fe concentrations. This phenomenon may be attributed to structural variations in the oxide layer of the surface layer.

Notably, distinct enrichment zones of Cl and Cu elements were detected in the corrosion products of three Cu-bearing 3.5%Ni weathering steels after being subjected to 72 h periodic immersion and a dry–wet cycle accelerated corrosion test. It was found that the Cl-rich region and the Cu-rich region were not in the same area. This phenomenon indicates that the oxide layer of the surface layer in the Cu-rich area was dense enough to prevent the entry of Cl^−^, or that the oxide layer of the surface layer in the Cu-rich area had ionic selectivity and could effectively repel Cl^−^. It is clear that no matter which mechanism is used, this is one of the reasons why Cu improves the corrosion resistance of the three Cu-bearing 3.5%Ni weathering steels. Different from the distributions of Fe, O, Cl, and Cu, in the oxide layer of the surface layer of the 72 h cycle immersion dry–wet cycle accelerated corrosion samples, both Ni and Mo maintain relatively uniform distributions throughout the oxide layer of the surface layer without observable localized enrichment. This homogeneous distribution pattern implies different corrosion inhibition mechanisms for these alloying elements compared to Cu, potentially involving more uniform electrochemical processes or oxide formation characteristics.

#### 3.2.5. Rust Structure Analysis

**Figure 6:**
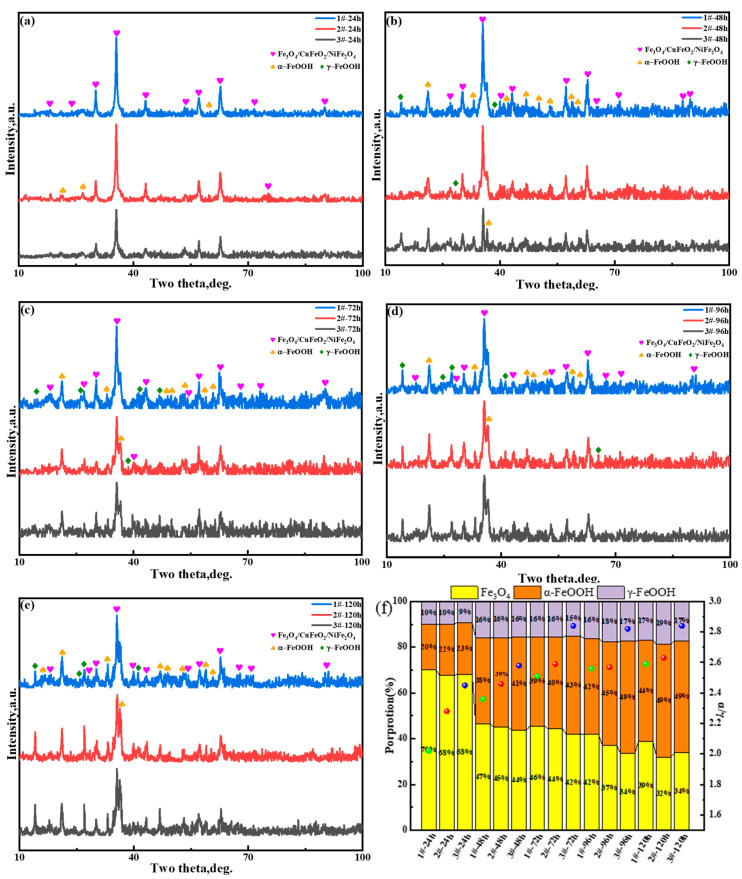
XRD patterns of rust powder of 3.5%Ni weathering steels after being subjected to accelerated dry–wet cycle corrosion tests for (**a**) 24 h, (**b**) 48 h, (**c**) 72 h, (**d**) 96 h, and (**e**) 120 h. (**f**) Fractions of corrosion products and α/γ value.

In order to further understand the influence of the Cu content on the component and phase composition of the oxide layer of the surface layer, periodic immersion dry–wet cycle accelerated corrosion tests of 3.5%Ni weathering steel were conducted for 24 h, 48 h, 72 h, 96 h, and 120 h. Then, the rust powder on the surface of the corroded samples was stripped off, and XRD analysis tests were carried out, respectively. It can be seen from the XRD spectra of each test steel under different corrosion cycles in Figure 6 that the phase types mainly include iron hydroxyl oxide (α-FeOOH and γ-FeOOH) and spinel phases (Fe_3_O_4_, CuFeO_2_, and NiFe_2_O_4_), and different phase types are distinguished by different color pattern types. Since the spinel phase diffraction peaks composed of Fe_3_O_4_, CuFeO_2_, and NiFe_2_O_4_ almost overlap and cannot be effectively distinguished, a pattern is used for marking.

Generally, Fe will generate different Fe-containing hydroxyl oxides and oxides under different conditions and environments. For example, the XRD patterns contain α-FeOOH, γ-FeOOH, and Fe_3_O_4_, all of which are common rust structures [37]. α-FeOOH and γ-FeOOH are isomers of each other. In terms of morphological differences, α-FeOOH is usually needle-like, while γ-FeOOH is rod-like [38]. The Gibbs free energies of α-FeOOH and γ-FeOOH are −775.76 KJ/mol and −709.52 KJ/mol, respectively (at room temperature), so α-FeOOH has higher stability [39]. α-FeOOH and γ-FeOOH also have different effects on the corrosion rate. α-FeOOH is a fine, needle-like and stable phase, which is more conducive to isolating Cl^−^ outward [40] and is beneficial for slowing down the corrosion of material. However, γ-FeOOH, as a coarse, rod-like active phase, has a relatively insignificant effect in slowing down the corrosion of material.

In order to quantify the protection ability of the oxide layer of the surface layer, the protection factor of the oxide layer of the surface layer is introduced. Yamashita [41] defined the protection factor of the oxide layer of the surface layer as α/γ (the mass ratio of α-FeOOH to γ-FeOOH), Kamimura [42] defined the protection factor of the oxide layer of the surface layer as α/γ* (the mass ratio of α-FeOOH to γ-FeOOH + β-FeOOH + Fe_3_O_4_), and Dillmann [43] defined the protection factor of the oxide layer of the surface layer as α*/γ* (the mass ratio of α-FeOOH + Fe_3_O_4_ and γ-FeOOH + β-FeOOH). Due to the short duration of the periodic dry–wet cycle accelerated corrosion test in the present work, the β-FeOOH substance could not be found under the environmental conditions of NaCl corrosion solution [44,45]. Therefore, α/γ is employed as the protection factor of the oxide layer of the surface layer for the protective ability.

For the same 3.5%Ni weathering steel, as the corrosion time increased, it could be clearly seen that the relative strength of the main peak at approximately 2θ = 35.5° (spinel phase) significantly decreased. With the increase in the Cu content, it could be seen that the intensity of the main peak at 2θ = 35.5° (spinel phase) decreased. According to the calculation principle of the half-height width of the X-ray diffraction formula [46], the reduction in the peak value indicates that the phase has low crystallinity. This confirms that with the increase in the Cu content, the content of α-FeOOH was significantly enhanced, the oxide layer of the surface layer strengthened the blocking effect on corrosive Cl^−^, and the corrosion resistance of 3.5%Ni weathering steel was improved. To accurately assess the protective capacity of the oxide layer of the surface layer, the mass percentages of each phase were determined and are shown in Figure 6f. Among them, the value of α/γ is positively correlated with the stability of the oxide layer of the surface layer [47], and the larger the value, the higher the stability of the oxide layer of the surface layer. It can be seen that under the accelerated corrosion tests of the same period of dry–wet cycles, the α/γ value increased with the increase in the Cu content. Under a corrosion time of 24 h, the α/γ value was about 2.02, 2.27, and 2.45 for the three steels. As the corrosion time increased to 120 h, the α/γ value increased to 2.58. 2.63, and 2.84. It was further confirmed that an increase in the Cu content could improve the oxide layer stability of 3.5%Ni weathering steel.

**Figure 7:**
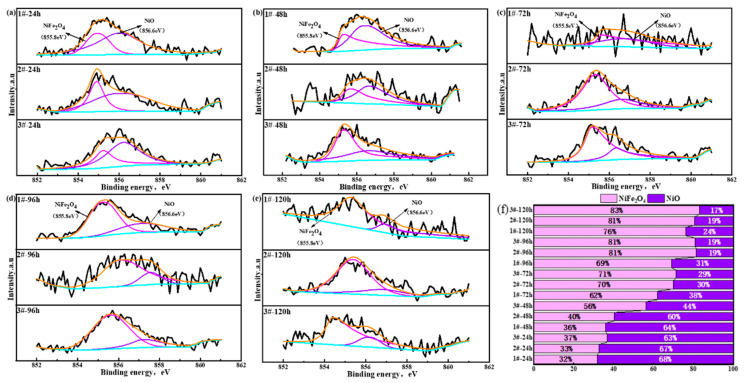
XPS spectrum of Ni-bearing compounds in rust powder of Cu-bearing weathering steels after dry–wet cycle accelerated corrosion tests for (**a**) 24 h, (**b**) 48 h, (**c**) 72 h, (**d**) 96 h, and (**e**) 120 h. (**f**) Fractions of Ni-bearing compounds.

**Figure 8:**
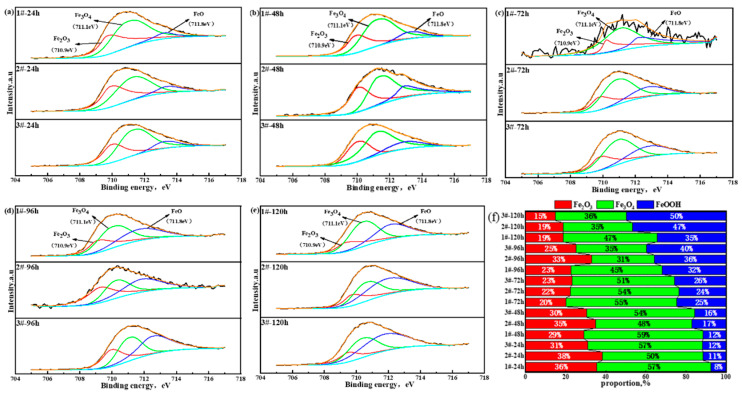
XPS spectrum of Fe-bearing compounds in rust powder of Cu-bearing weathering steels after dry–wet cycle accelerated corrosion tests for (**a**) 24 h, (**b**) 48 h, (**c**) 72 h, (**d**) 96 h, and (**e**) 120 h. (**f**) Fractions of Fe-bearing compounds.

**Figure 9:**
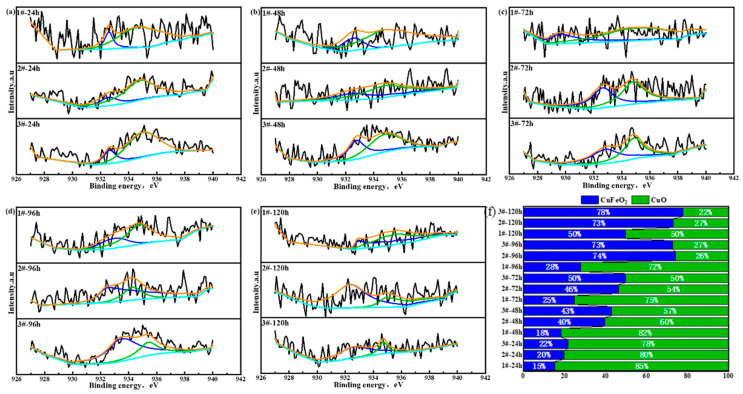
XPS spectrum of Cu-bearing compounds in rust powder of Cu-bearing weathering steels after dry–wet cycle accelerated corrosion tests for (**a**) 24 h, (**b**) 48 h, (**c**) 72 h, (**d**) 96 h, and (**e**) 120 h. (**f**) Fractions of Cu-bearing compounds.

Since XRD reflects the crystal structure information of the rust constituents, it could not obtain the basic information of different valence states of the elements in the constituent substances. In order to further understand the existence forms of valence states of Fe, Ni, and Cu in each Cu-bearing weathering steel, periodic immersion dry–wet cycle accelerated corrosion tests of 3.5%Ni weathering steel with different Cu contents were conducted for 24 h, 48 h, 72 h, 96 h, and 120 h. Then, the rust powder of the corroded samples was stripped off, and an XPS test analysis was carried out, respectively. The main elements (Ni, Fe, and Cu) in each 3.5%Ni weathering steel were selected for fine spectral analysis. The binding energies of each compound were found through reference data periodic tables such as the peak position and peak width of the XPS binding energy [48] and relevant queries in the literature, as shown in Table 3. Figure 7, Figure 8 and Figure 9 display the XPS spectra of Ni, Fe, and Cu in rust powder of Cu-bearing 3.5%Ni weathering steel after 24 h, 48 h, 72 h, 96 h, and 120 h periodic immersion dry–wet cycle accelerated corrosion tests, respectively. The results indicate that the existence forms of Fe elements are Fe_2_O_3_, Fe_3_O_4_, and FeOOH. The existence forms of Ni are NiO and NiFe_2_O_4_, which have been reported previously [49]. Similarly, the existence forms of Cu are CuO and CuFeO_2_, and the composition system also fits well with the results of Hao et al. [50].

An XPS semi-quantitative analysis was conducted. The valence state ratios obtained through the valence state areas of Ni, Fe, and Cu are shown in Figure 7f, Figure 8f and Figure 9f, respectively. It could be known from the proportion of Ni valence states in Figure 7f that the content of Ni compounds was related to the variation in the Cu content. This is because with the increase in the Cu content, the proportion of NiFe_2_O_4_ increased and the proportion of NiO decreased. NiFe_2_O_4_, as an electronegative substance [51], can concentrate Cl elements and block them outside of the substrate, protecting the stability of the oxide layer of the surface layer.

An Fe valence state ratio diagram is displayed in Figure 8. With the increase in the accelerated corrosion time of the periodic dry–wet cycle, the percentages of the FeOOH phase in each corrosion steel increased because more α-FeOOH appeared. However, Fe_3_O_4_, as a conductor, serves as a natural channel for electrons, which is not conducive to the improvement in corrosion resistance. Both the proportion decrease in the Fe_3_O_4_-phase content and proportion increase in the α-FeOOH-phase content correspond to the decrease in the corrosion rate of Cu-bearing weathering steel, which is the same as the thickness loss result in Figure 1.

The Cu valence state ratio diagram regarding Cu-containing compounds is displayed in Figure 9. With the increase in both the Cu content and corrosion time, the proportion of CuFeO_2_ significantly increased. Referring to the common chemical thermodynamic property table [52], it is seen that at room temperature, the thermodynamic property stability of CuFeO_2_ with its Gibbs free energy (−4.799 × 10^5^ J/mol) is higher than that of Fe_3_O_4_ (−1.015 × 10^6^ J/mol).

#### 3.2.6. Electrochemical Test Analysis (Polarization Curves)

When the content of Cl^−^ in the corrosion solution increases, the number of ions undergoing directional movement increases, resulting in an increase in the conductivity of the solution and, subsequently, an accelerated corrosion rate [53]. Generally, in systems where NaCl solution is used as the corrosive solution, the corrosion rate is also determined by factors such as the pH value, solution temperature, and the limiting diffusion current density for reduction by dissolved oxygen. As the content of Cl^−^ in the solution increases, it will occupy the position of O_2_ in the solution, thereby leading to a gradual decrease in the solubility of O_2_ in the solution [54]. Studies have proven that when the concentration of NaCl solution is 2%wt, its comprehensive corrosion rate is the best, and the solution system is also more stable. In addition, a constant temperature box maintaining the temperature of the NaCl solution system at 35 °C is set up to ensure it is close to the temperature of the simulated South China Sea test and to maintain the neutral range of the pH value of the NaCl solution system.

**Figure 10:**
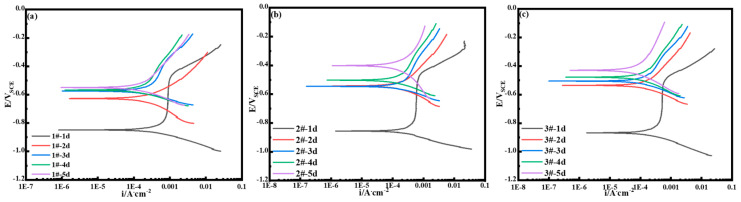
Polarization curve of steel under accelerated dry–wet cycle corrosion test: (**a**) 1#-0.75Cu steel; (**b**) 2#-1.25Cu steel; and (**c**) 3#-2.15Cu steel.

The dynamic potential polarization curves of all 3.5%Ni weathering steels are shown in Figure 10. The polarization curves of each 3.5%Ni weathering steel at 24 h show obvious passivation phenomena. This is because in the early stage of corrosion, the sample surface has not completely rusted. Therefore, polarization behavior is affected by the combined action of the matrix and oxide film. With the extension of the periodic immersion corrosion test, it can be seen that the thickness of the oxide layer of the surface layer and the change in the polarization curve are dominated by the oxide layer of the surface layer. During the period from 48 h to 120 h, the polarization curve shapes of all steels were similar, which means that after the 48 h periodic wet–dry cycle accelerated corrosion test, their electrochemical mechanisms did not change. The anode part is the active dissolution of steel, and the cathode part is mainly the oxygen reduction process. The change in the Cu content in 3.5%Ni weathering steel will affect the electrochemical activity, and the polarization curve data measured by electrochemistry are fitted by Tafel. Both the current density (Icorr) and corrosion potential (Ecorr) are shown in Table 4 and Table 5.

**Figure 11:**
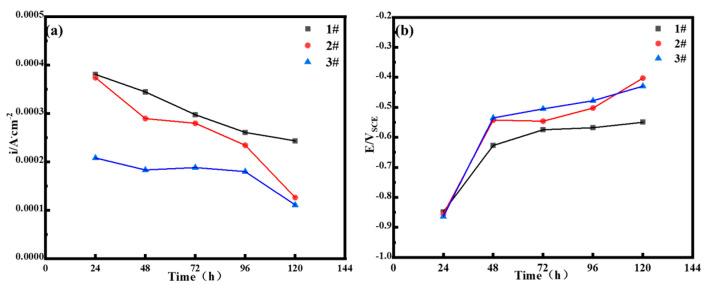
Relationship of corrosion potential (**a**) and current density (**b**) changes with corrosion time.

Based on Table 4 and Table 5, the relationship of the current density and corrosion potential with the corrosion test cycle was determined and is shown in Figure 11. Under the same corrosion period, for 3.5%Ni weathering steel, as the Cu content increased, the corrosion current decreased and the corrosion potential shifted positively, indicating that the anodic process was inhibited to a certain extent. This suggests that an increase in the Cu content in solution state would improve the corrosion resistance of the oxide layer of the surface layer when 3.5%Ni weathering steel is subjected to corrosion in the South China Sea.

## 4. Discussions

Cu, a key alloying element in expanding the austenite phase region, significantly enhances the stability of undercooled austenite, thereby influencing the phase transformation behavior and microstructural characteristics of steel. The addition of an appropriate amount of Cu in the 3.5Ni steel system not only improves the overall corrosion resistance through solid solution strengthening—particularly enhancing resistance to uniform and localized corrosion in acidic environments—but also effectively increases hardenability, promoting bainitic transformation while suppressing the formation of proeutectoid ferrite. This phenomenon is clearly demonstrated in the experimental results shown in Figure 1: a microstructural analysis reveals that with a gradual increase in the Cu content (0.75–2.15 wt%), there is a monotonic decrease in the ferrite phase fraction, accompanied by a corresponding increase in the bainite volume fraction. Notably, Cu addition induces significant grain refinement, primarily attributed to its pinning effect on austenite grain boundaries and its inhibitory role in phase transformation nucleation during the cooling process. This microstructural optimization leads to a remarkable improvement in strength while maintaining good toughness, providing critical theoretical insights and practical guidance for the development of advanced high-performance low-alloy steels.

Further mechanical property investigations revealed that an enhanced bainite content leads to a continuous improvement in strength with an increasing Cu concentration. However, this strengthening effect is accompanied by a reduction in low-temperature impact toughness, particularly pronounced in the ductile-to-brittle transition temperature range. An in-depth analysis indicates that this toughness deterioration stems from two primary factors: firstly, solute Cu atoms tend to segregate at grain boundaries during cooling, reducing grain boundary cohesion energy and increasing embrittlement susceptibility; secondly, the potential precipitation of ε-Cu phases during aging may further exacerbate material embrittlement. However, as the precipitation temperature range was avoided, there was no ε-Cu nano-scale precipitation phase. This strength–toughness trade-off relationship necessitates careful optimization of the Cu content in practical engineering applications. An appropriate microalloying design and heat treatment process adjustments are required to mitigate Cu-induced embrittlement tendencies, thereby maintaining satisfactory low-temperature toughness while ensuring material strength to meet demanding service requirements in extreme environments.

When copper (Cu) exists in solid solution state within steel, its effect on improving corrosion resistance is particularly remarkable. Experimental data demonstrate that as the Cu content increases from 0.75 wt% to 1.25 wt%, the corrosion rate of steel in simulated corrosive environments shows a significant decreasing trend, with a reduction exceeding 20%. This improvement is primarily attributed to the solid solution Cu promoting the formation of a denser passive film on the material surface and inhibiting localized corrosion through cathodic protection effects. However, when the Cu content further increases to 2.15 wt%, the change in corrosion rate tends to stabilize, indicating a clear concentration saturation effect of solid solution Cu on corrosion resistance enhancement.

With the increase in the Cu content in 3.5%Ni steel, the content of α-FeOOH was significantly enhanced. The oxide layer of the surface layer strengthened the blocking effect on corrosive Cl^−^, and the corrosion resistance of 3.5%Ni weathering steel was improved. Under the corrosion time of 24 h, the α/γ value was about 2.02, 2.27, and 2.45 for 0.75Cu, 1.25Cu, and 2.15Cu steels. As the corrosion time increased to 120 h, the α/γ value increased to 2.58. 2.63, and 2.84. It was further confirmed that the increase in the Cu content could improve the oxide layer stability of 3.5%Ni weathering steel. Further XPS experimental results confirmed that the corrosion-resistant mediua that prevent Cl^−^ are α-FeOOH, NiFe_2_O_4_, CuO, and CuFeO_2_.

Notably, this concentration dependence exhibits a significant difference from the influence pattern of Cu on impact toughness: even in higher Cu content ranges, its detrimental effect on toughness continues to intensify. This differentiated mechanism suggests that Cu’s improvement in corrosion resistance mainly stems from its surface electrochemical behavior, while its impact on toughness is primarily related to its segregation behavior at grain boundaries, representing two entirely distinct mechanisms. This finding provides important insights for subsequent alloy design: while pursuing optimal corrosion resistance, material toughness requirements must also be considered. By precisely controlling the Cu content within the optimized range of 1.0–1.5 wt%, the best performance balance can be achieved.

## 5. Conclusions

The effect of the Cu content on the corrosion resistance of 3.5%Ni marine atmospheric weathering steel was investigated by microstructural analysis, mechanical property tests, and a series of corrosion tests. The main conclusions are as follows:

(1) The microstructure of 0.75Cu steel involves bainite plus ferrite, while the microstructure of 1.25Cu and 2.15Cu steels involves bainite. With the increase in the Cu content, the strength of 3.5%Ni steel increased, while the impact toughness at −20 °C decreased from 96 J (0.75Cu steel) to 13 J (2.15Cu steel).
(2) Under the simulation of marine atmospheric corrosion in the South China Sea by periodic dry–wet cycle accelerated corrosion tests, the corrosion resistance of 3.5%Ni weathering steel increased accordingly with the increase in the Cu content. The polarization curves of the rusted samples show that corrosion potential shifts positively and corrosion current density decreases.
(3) As the Cu content increased, the surface roughness of the 3.5%Ni weathering steel after corrosion decreased, and the corrosion degree was lower. With a higher Cu content, the corrosion behavior was more uniform, which is beneficial to the formation of a uniform oxide layer at the surface layer.
(4) After the 72 h periodic immersion dry–wet cycle accelerated corrosion test, there were different Cl-rich areas and the Cu-rich areas in the oxide layer of the surface layer. The Cu-rich areas involved CuO and CuFeO_2_, significantly inhibiting the penetration of Cl^−^.
(5) After accelerated corrosion tests, the phase structures of the oxide layer of the surface layer mainly included two categories: iron hydroxide oxide (α-FeOOH and γ-FeOOH) and spinel phases (Fe_3_O_4_, CuFeO_2_, and NiFe_2_O_4_). With the increase in the Cu content, the fraction of α-FeOOH significantly increased, and the value of the α/γ protective factor of the oxide layer of the surface layer became larger.

## 6. Limitations and Challenges

The results obtained in this study are all based on the laboratory simulation of accelerated corrosion tests. Although the atmospheric environment of the South China Sea was simulated by strictly controlling key parameters such as the temperature, humidity, and Cl^−^ concentration, there are still certain differences from actual marine atmospheric corrosion conditions. To obtain research data with greater engineering guidance value, future research work will focus on in-depth explorations involving the following: (1) An atmospheric exposure test station will be established in typical sea areas of the South China Sea, and a 2–5 year real-sea hanging test will be conducted to systematically investigate the corrosion behavior law of Cu-containing steel in the actual marine atmospheric environment. (2) Based on the microclimate characteristics of different islands in the South China Sea (such as monsoon variations, the frequency of dry–wet alternations, salt spray deposition, etc.), a more accurate model of environment–material coupling effects will be established. (3) For the optimized design of the Cu content, the gradient will be further refined on the basis of existing research. More precise content gradients such as 0.5%, 0.8%, 1.0%, 1.2%, and 1.5% should be set, and the influence mechanism of trace Cu (<0.3%) on the corrosion resistance of steel will be studied. (4) Simultaneously, research on the synergistic effect of Cu elements with other alloying elements (such as Ni, Cr, Mo, etc.) will be carried out to provide a theoretical basis for the development of new weathering steel suitable for the special environment of the South China Sea. These research efforts will help establish a reliable connection between laboratory data and practical engineering applications.

## Figures and Tables

**Table 1:** Nominal chemical composition of tested steel (wt.%).

Specimen	C	Si	Mn	P	S	Ni	Mo	Cu
1# (0.75Cu)	0.020	0.19	0.74	<0.01	<0.001	3.56	0.25	0.75
2# (1.25Cu)	0.019	0.21	0.73	<0.01	<0.001	3.55	0.25	1.25
3# (2.15Cu)	0.022	0.2	0.73	<0.01	<0.001	3.55	0.25	2.15

**Table 2:** Mechanical properties of tested steel.

Specimens	Impact Energy at RT/J	Impact Energy at −20 °C/J	TS/MPa	YS/MPa	TE/%	RA/%
1#	99	96	680	560	21	77
2#	104	68	724	651	21	72
3#	25	13	822	713	20	75

YS: yield strength; TS: tensile strength; RT: room temperature; TE: total elongation; RA: reduction in area.

**Table 3:** Main compounds in oxide layer of surface layer and binding energy (eV).

Main Compounds in Oxide Layer of Surface Layer and Binding Energy (eV)				
Ni 2p3/2	Peak	NiFe_2_O_4_	NiO	
	EB/eV	855.8	856.6	
Fe 2p3/2	Peak	Fe_2_O_3_	Fe_3_O_4_	FeOOH
	EB/eV	710.9	711.1	711.8
Cu 2p3/2	Peak	CuFeO_2_	CuO	
	EB/eV	932.6	934.8	

**Table 4:** Current density of Cu-bearing 3.5%Ni steel under accelerated dry–wet cycle corrosion tests (1 × 10^−4^ A/cm^2^).

Samples	24 h	48 h	72 h	96 h	120 h
1#	6.8039	3.4432	2.9724	2.6072	2.4321
2#	3.7376	2.8933	2.7949	2.3413	1.2609
3#	2.079	1.8313	1.8802	1.7967	1.1080

**Table 5:** Corrosion potential of Cu-bearing 3.5%Ni steel under accelerated dry–wet cycle corrosion tests (V).

Samples	24 h	48 h	72 h	96 h	120 h
1#	−0.84813	−0.62675	−0.5745	−0.56769	−0.54944
2#	−0.85544	−0.54231	−0.5460	−0.50237	−0.40243
3#	−0.86328	−0.53506	−0.5050	−0.47775	−0.42931

## Data Availability

The original contributions presented in this study are included in the article. Further inquiries can be directed to the corresponding author.
